# Early administration of L‐arginine in *mdx* neonatal mice delays the onset of muscular dystrophy in tibialis anterior (TA) muscle

**DOI:** 10.1096/fba.2020-00104

**Published:** 2021-05-18

**Authors:** Roy W. R. Dudley, Alain S. Comtois, David H. St‐Pierre, Gawiyou Danialou

**Affiliations:** ^1^ Meakins Christie Laboratories McGill University Montreal QC Canada; ^2^ Département des Sciences de l’Activité Physique Université du Québec à Montréal (UQAM Montreal QC Canada; ^3^ Groupe de Recherche en Activité Physique Adaptée UQAM Montreal QC Canada; ^4^ Centre de Recherche du CHU Sainte‐Justine Montréal QC Canada; ^5^ Royal Military College Saint‐Jean Saint‐Jean‐sur‐Richelieu QC Canada

**Keywords:** L‐arginine, *mdx*, neonates, nitric oxide, utrophin

## Abstract

Duchenne muscular dystrophy (DMD) is a genetic disorder that results in the absence of dystrophin, a cytoskeletal protein. Individuals with this disease experience progressive muscle destruction, which leads to muscle weakness. Studies have been conducted to find solutions for the relief of individuals with this disease, several of which have shown that utrophin, a protein closely related to dystrophin, when overexpressed in *mdx* neonatal mice (the murine model of DMD), is able to prevent the progressive muscle destruction observed in the absence of dystrophin. Furthermore, recent studies have shown that L‐arginine induces utrophin upregulation in adult *mdx* mice. We hypothesized that L‐arginine treatment also induces utrophin upregulation to prevent the development of muscle weakness in neonatal *mdx* mice. Hence, L‐arginine should also prevent progressive muscle destruction via utrophin upregulation in *mdx* neonatal mice. *Mdx* neonatal mice were injected intraperitoneally daily with 800 mg/kg of L‐arginine for 6 weeks, whereas control mice were injected with a physiological saline. The following experiments were performed on the tibialis anterior (TA) muscle: muscle contractility and resistance to mechanical stress; central nucleation and peripheral nucleation, utrophin, and creatine kinase quantification as well as a nitric oxide (NO) assay. Our findings show that early administration of L‐arginine in *mdx* neonatal mice prevents the destruction of the tibialis anterior (TA) muscle. However, this improvement was related to nitric oxide (NO) production rather than the expected utrophin upregulation.

**Abbreviations**
BSA: bovine serum albumin
CK: creatine kinase
DMD: duchenne muscular dystrophy
EDL: extensor digitorium longus
eNOS: endothelial nitric oxide synthase
H&E: hematoxylin–eosin
IgG: immunoglobulin G
iNOS: inductible nitric oxide synthase
IP: intraperitoneal
Lo: optimal length
min: minute
ms: milliseconds
nNOS: neuronal nitric oxide synthase
NO: nitric oxide
PBS: phosphate‐buffered saline
TA: tibialis anterior
TBS‐T: tris‐buffered saline tween 20

## 1 INTRODUCTION

Duchenne muscular dystrophy (DMD) is a genetic disorder that affects one in 3500 males at birth. In individuals with this disease, as well as in *mdx* mice (the murine model of DMD), the absence of dystrophin, a cytoskeletal protein, is observed. In DMD patients, the progressive destruction of muscle is associated with fibrosis and intracellular lipid infiltration, resulting in muscle weakness and the death of affected individuals around the age of 30.^1^ In normal muscles, dystrophin is associated with a group of proteins, as well as the multifunctional enzyme, neuronal nitric oxide synthase (nNOS). The complex formed by dystrophin and these proteins forms a physical link between the extracellular matrix and the internal cytoskeleton that is supposed to stabilize the sarcolemma and thus prevent damage resulting from mechanical stress, such as muscle contractions.^2^, ^3^, ^4^ It should also be noted that the absence of dystrophin results in a secondary loss or reduction of its associated proteins.^3^, ^5^ As such, *mdx* mouse muscles are abnormally susceptible to damage from mechanical stress.^3^, ^6^, ^7^

Data suggest that L‐arginine treatment promotes utrophin overexpression in *mdx* adult mice.^8^, ^9^ In turn, this effect is thought to improve the physiology of the diaphragm and the *extensor digitorium longus* (EDL) muscles.^9^, ^10^ Utrophin is a protein naturally produced in both humans and animals during muscle development, repair, and regeneration. When non‐dystrophic skeletal muscle matures, utrophin is replaced by dystrophin.^11^, ^12^, ^13^ Several studies, however, have shown that the lack of functional dystrophin (such as in DMD and *mdx* mice) can be compensated by utrophin expression, and this compensation can promote skeletal muscle performance.^14^, ^15^, ^16^, ^17^, ^18^ Hence, in the absence of dystrophin (such as in DMD), utrophin overexpression may play a protective role in the maintenance of sarcolemma integrity.^14^, ^15^, ^16^, ^17^, ^18^ However, despite being considered a strong candidate to restore skeletal muscle functions in DMD, there is at least one of dystrophin's critical functions that utrophin is unable to perform.^19^ Utrophin, unlike dystrophin, lacks the capacity to anchor the nNOS protein to the cell membrane of the skeletal muscle.^19^ Hence, the absence of this nNOS anchoring capacity to the sarcolemma strongly impairs the potential use of utrophin as a pharmacological candidate to treat DMD. Nitric oxide (NO) is an endogenously synthesized molecule performing ubiquitous physiological activities in living organisms.^20^, ^21^, ^22^ NO is produced from its biological precursor, L‐arginine, through at least three different pathways: eNOS (endothelial cells), nNOS (neuronal cells), and iNOS (in response to immune activation).^23^ NO is also thought to play an important role in the regulation of physio‐pathological processes.^24^ In skeletal muscle physiology, NO is an important signaling molecule^25^ particularly for the processes relating to adaptation from exercise.^26^ Indeed, in normal skeletal muscle, NO regulates vascular perfusion during muscle contraction, maintains mitochondrial functions, and modulates glucose uptake and excitation‐contraction coupling.^27^, ^28^ All three NOS isoforms (nNOS, eNOS, and iNOS) are present in skeletal muscle tissue, with nNOS, however, being predominant.^29^, ^30^ It should be noted that it is primarily eNOS which is present in endothelial cells in the vasculature of skeletal muscle. However, some reports show low levels of eNOS, and iNOS in skeletal muscle. In dystrophic muscle tissue, high levels of iNOS are found due to the presence of inflammatory cells.^25^, ^27^, ^28^, ^31^, ^32^, ^33^

Based on the aforementioned evidence, the purpose of this study was to determine the effects of exogenous L‐arginine treatment on the stimulation of utrophin protein expression in *mdx* neonatal skeletal muscles. Indeed, the previous experiments regarding NO and utrophin were performed only on adult *mdx* mice. The hypothesis was that exogenous L‐arginine would overexpress utrophin in neonatal *mdx* muscle. This overexpression could promote positive outcomes on skeletal muscle functions in newly born mice and thus delay or prevent the onset of DMD.

## 2 MATERIALS AND METHODS

### 2.1 Animal procedures

All procedures on mice were performed according to the McGill University Guidelines for Animal Care (protocol 2001–3480). An Intraperitoneal (IP) injection of L‐arginine (800 mg/kg of body weight diluted in sterile physiological saline) or sterile physiological saline alone (control) was performed on 7‐day‐old *mdx* neonatal mice (C57/BL10ScSn‐*mdx* J; Jackson Laboratory, Bar Harbor, ME, USA). The *mdx* neonatal mice receiving L‐arginine or saline were injected daily for 6 weeks. Mice were weighed daily throughout the experiments. Only *mdx* male mice were selected for the experiments. A randomized double‐blinded protocol was used.

### 2.2 In vivo measurement of TA contractility

The contractility experiments were performed as previously described.^3^, ^34^, ^35^ Briefly, after 6 weeks of treatment, mice were anesthetized with a mixture of ketamine (130 mg/kg) and xylazine (20 mg/kg) and immobilized in the supine position on a surgical platform. The legs were stabilized using two needles (to secure the knees and ankles). An incision was performed in the skin to expose the TA muscle. The distal tendon was isolated and tied with silk suture to a force transducer (Model 305B dual mode; Aurora Scientific, Cambridge Technology, Watertown, MA, US). An electrode was then connected to a Grass stimulator (Model SS44; Grass Instruments, Quincy, MA, USA) and placed on the muscle, which was constantly irrigated with a physiological saline solution heated to 37ºC. The muscle force and length were measured using Ladat/Anadat software (RHT‐Info Data, Montreal, Quebec, Canada).

#### 2.2.1 Measurement of the muscle force‐generating capacity

After obtaining the optimal length (Lo) of the muscle (i.e., the muscle length that generates the maximal force with a single stimulation), the muscle was stimulated at the following frequencies: 10, 30, 60, 90, and 120 Hz for 300 ms, with 2‐min intervals between each stimulation. As a result, 120 Hz was found to represent the stimulation frequency at which the maximal isometric tetanic force was measured.^3^, ^34^

#### 2.2.2 Eccentric contraction

After the last stimulation at 120 Hz and the 2‐min recovery period, five consecutive eccentric contractions were performed at 120 Hz with intervals of 30 s between each stimulation. The muscle was stimulated for a total of 300 ms; during the first 100 ms, the muscle was held at Lo (isometric component) but lengthened through 25% of the optimal length during the last 200 ms (eccentric component). The final level of isometric force production was performed at the end of the five eccentric contractions at 120 Hz. At the end of the experiment, muscle weight and length (Lo) were measured in order to obtain the specific force (N/cm^2^) from each muscle.^3^, ^35^

### 2.3 Utrophin immunostaining, quantification of the number of fiber and nucleation, and muscle cross‐sectional area measurement

Since mechanical stress induces muscle damage, one TA from each animal was used for the contractility experiments and utrophin western blotting, whereas the contralateral TA was used for staining (hematoxylin‐eosin/H&E and immunostaining). TA muscle was mounted in frozen section medium (Stephens Scientific, Riverdale, NJ, USA) and snap‐frozen in methyl butane pre‐cooled with liquid nitrogen. Superfrost slides were used to collect the transverse cryostat sections (5 µm) from the midportion of the frozen muscle. Immunostaining was performed as follows: muscle sections were rehydrated with phosphate‐buffered saline (PBS) solution and blocked with a mix of 3% Bovine serum albumin (BSA) +3% goat serum. After 30 min, muscle slices were blocked with affinity‐purified goat anti‐mouse IgG Fab fragment (Jackson ImmunoReseach, West Grove, PA, USA) followed by incubation with a primary antibody against utrophin (Novocastra Laboratories, Newcastle‐upon‐Tyne, UK) for 1 h. After washing the slides three times (5‐min washes) with PBS, muscle sections were incubated with a biotinylated goat F(Ab’)2 anti‐mouse (Southern Biotech, Birmingham, AL, USA) followed by a reaction with Cy3‐conjugated streptavidin (Jackson ImmunoReseach, West Grove, PA, USA). Muscle sections without the primary antibody were used as controls. To evaluate the number of fibers per muscle section, and the detection of cells with nucleation (central and peripheral nucleation), the TA sections were counterstained using H&E staining.^36^, ^37^ Central nucleation is an indicator of necrosis and muscle regeneration. Stained muscle sections were visualized using fluorescence microscopy and photographed using a digital camera. Analysis of the number of utrophin‐positive myofibers, the muscle cross‐sectional area measurement, the number of fibers, and cells with central and peripheral nucleation was performed on entire muscle cross sections. The number of individual muscle fibers was manually counted (manual tag) using a commercial software package (Image‐Pro Plus; Media Cybernetics, Silver Spring, MD). Muscle regeneration was analyzed by calculating the percentage of fibers with central nucleation relative to the total number of fibers.^38^ The same strategy was used for determining the number of utrophin‐positive muscle fibers, and the percentage of fibers with peripheral nucleation. It should be noted that only muscle fibers presenting utrophin staining over their entire circumference were considered. For the muscle cross‐sectional area measurement, digital imaging was performed at 4x final magnification. The circumference of each muscle section was outlined using Image‐Pro Plus software to generate the muscle cross‐sectional area. Image analyses were performed by two observers and were averaged.

### 2.4 Utrophin western blotting

Utrophin western blotting was performed as previously described, according to the standard method.^39^, ^40^, ^41^ In our experiments, western blotting was systematically performed on the supernatant and cell pellet. Briefly, 50 cryostat sections at 20 µm were collected from each muscle and incubated for 20 min in 100 µl ice‐cold buffer A (0.1 M KH_2_PO_4_, 0.05 M K_2_HPO_4_, 5 mM MgCl_2_, 5 mM ATP, 0.3 M KCl, pH 6.5) followed by the addition of fresh protein inhibitors (1 mM PMSF, 2.5 U/ml Aprotinin, 0.5 µg/ml Leupeptin). During the incubation period, the samples were sonicated and centrifuged for 15 min at 15,000 *g* and 4ºC. The supernatants were collected and kept on ice while the cell pellets were resuspended in 30 µl of loading buffer (62 mM Tris HCl pH 6.8, 10% glycerol, 2% sodium dodecyl sulfate). The supernatant and the resuspended pellets were centrifuged for 10 s before quantifying the proteins using Bradford assay. Next, 15 µg of total protein from the supernatant and 10 µg of total protein from the pellet were mixed with loading buffer (1× Laemmeli buffer containing 0.02% bromophenol blue +5% β‐mercaptoethanol) and boiled for 5 min at 100ºC before being rapidly centrifuged. Each sample was loaded in a 3% stacking gel (12.6 ml H_2_O, 5 ml of 0.5 M Tris pH 6.8, 2 ml Acryl bis 30%, 0.2 ml of 10% sodium dodecyl sulfate, 0.2 ml of 10% APS, 0.01 ml TEMED in total volume of 20 ml). The resolving gel was casted at 4% agarose. Protein migration was performed at 60 V in the running buffer until a clear line of migration appeared in the gel, at which point the voltage was increased to 100 V. The protein migration was stopped when the line had run off the gel. The transfer of the protein onto a nitrocellulose membrane was carried out overnight at 40 V. The membrane was stained with Ponceau S (0.005% in 1% acetic acid) to confirm that equal amounts of protein had been loaded, followed by blocking for 1 h in 0.1% TBS‐T containing 10% BSA under slight agitation. The membranes were incubated overnight with the primary antibody (mouse anti‐utrophin NCL‐DRP2 diluted at 1:200). Each membrane was then washed twice for 10 min with 0.1% TBS‐T. The goat anti‐mouse‐HRP diluted at 1:10,000 was then added to the membrane as the secondary antibody, incubated for 1 h at room temperature (22–24ºC). The membrane was washed three times. ECL plus was then added to the membrane for 1 min to develop the film. For equal loading, myosin protein was used. Densitometric quantification was calculated using the NIH ImageJ program. The average of three assays from each sample was used to compare the animals treated with L‐arginine with the control animals.

### 2.5 Measurement of NO production

The animals were treated for 6 weeks as previously described. Several methods for detecting NO release can be used: Griess assay, fluorescence, chemiluminescence, and electrochemical methods. In our experiment, NO production was measured by chemiluminescence, as described by Dudley et al.^42^ Briefly, the two TA muscles from each mouse were pooled, quickly ground in liquid nitrogen, homogenized in cold buffer, and centrifuged for 30 min. Each sample was adjusted to contain a uniform protein concentration of 8 µg/µl. Then, an equal volume of 40% trichloroacetic acid was added to prevent the foaming of the samples. The samples were centrifuged at 15,000 rpm for 10 min. Next, 40 µl of each sample was injected into a purge vessel containing the reducing agent, which allowed the nitrite and nitrate present in the samples to be reduced to NO, resulting in the production of a chemiluminescent signal.^43^ Various concentrations of sodium nitrate solution were used to produce a standard curve, which was in turn used to calculate the concentration of NO and its metabolites. Each sample was assayed in triplicate. NOx (NO and its metabolites) were quantified using a Sievers 280 NO Analyzer (NOA) (Sievers Instruments Inc., Boulder, CO).

### 2.6 Creatine phosphate kinase (CK) assay

The blood from the mice used for the measurement of NO production was used for the CK assay. Blood samples were collected before the harvest of the TA muscles for NO measurement. Blood was extracted from the femoral vein and stored in a fridge (4ºC) for 10 min. Blood samples were then centrifuged at 4ºC for 15 min at 15,000 *g* with IEC Micromax (IEC Micromax, Needham Heights, MA, USA). Serum was then collected to perform the CK assay. CK activity was measured by the colorimetric method using a commercial CK activity assay kit (ab155901. Abcam Inc. Toronto, ON, Canada).

### 2.7 Statistical analysis

The results are expressed as mean ± standard error (SE). Statistical analysis was performed using a statistics software package (SigmaStat, Jandel Scientific, San Rafael, CA, USA). Unpaired Student's *t*‐test for independent samples and multiple statistical comparisons between groups was performed by one‐way analysis variance (ANOVA) followed by Bonferroni's *t*‐test post hoc correction to allow for a better evaluation of the intra‐ and inter‐group variabilities and to prevent the inflation of the false‐positive rate. *p*<0.05 was considered as statistically significant.

## 3 RESULTS

Preliminary experiments were performed as described above in Animal procedures, with two doses: 200 mg, and 400 mg/kg of body weight of L‐arginine injected daily in seven days old mice for 6 weeks. After the treatment with L‐arginine, muscle contractility and resistance to mechanical stress were evaluated in the *tibialis anterior* (TA) muscle. Our preliminary results showed that baseline contractile functions (contractility and muscle resistance to mechanical stress) were not different between *mdx* neonatal mice treated with saline (control), and *mdx* neonatal mice treated with 200 mg or 400 mg/kg of L‐arginine (see Figures 3–10 in supplemental data). In addition, no difference was observed regarding total body or TA muscle weights between mice of the two groups (L‐arginine vs. saline). A dose of 800 mg/kg was thus chosen. This dose (doubling of the doses beginning at 400 mg/kg of body weight) in neonatal *mdx* mice did not result in any negative visible impact on external (e.g., skin at the injection site) or internal organs. In fact, the behaviors of the treated animals were not different from those of control animals (receiving physiological saline alone). The animals did not display any adverse effects after receiving daily intraperitoneal injections of L‐arginine.

### 3.1 Impact of L‐arginine treatment on body, TA muscle, and heart weights

**FIGURE 1:**
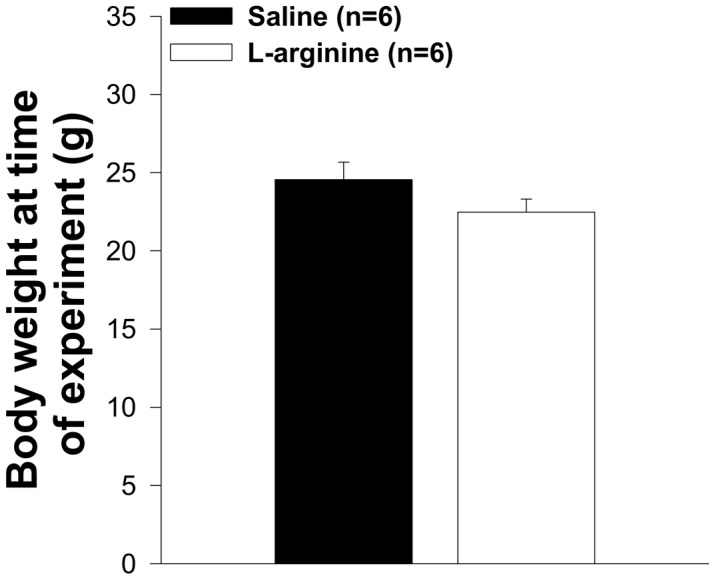
Body weight in L‐arginine and saline‐treated *mdx* mice. Body weights in both groups of animals are not different. There is no statistical difference between both groups of animals. Values represent group means ± standard error (SE); n represents the number of animals in each group

**FIGURE 2:**
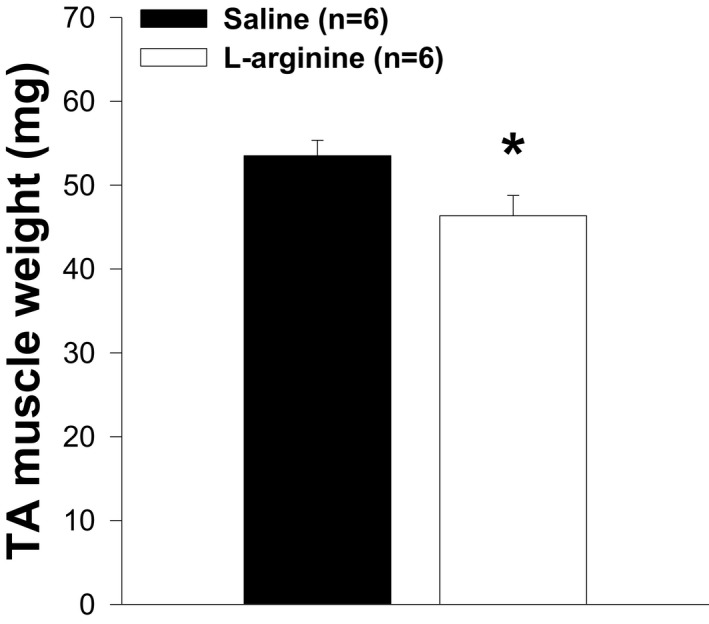
Tibialis anterior (TA) muscle weight in both groups of animals. TA muscle weight in *mdx* mice treated with L‐arginine is significantly lower than in the control group (*p<0.05), whereas the body weight is not different between the two groups of mice after 6 weeks of treatment. Values represent group means ± SE; n represents the number of TA muscles analyzed

**FIGURE 3:**
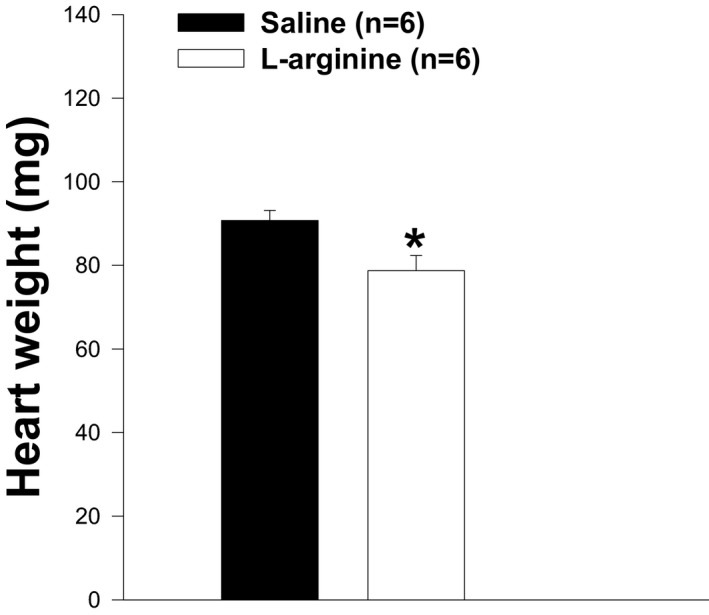
Heart weight in both groups of animals. Heart weight in *mdx* mice treated with L‐arginine is significantly lower than in control group (*p<0.05), whereas the body weight is not different between the two groups of mice after six weeks of treatment. Values represent group means ± SE; n represents the number of hearts analyzed

**FIGURE 4:**
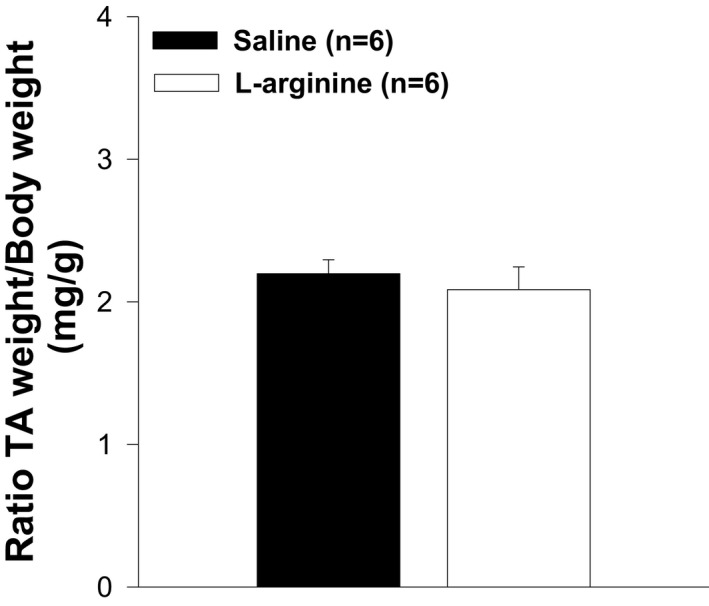
TA muscle weight normalized to the body weight in L‐arginine and saline‐treated *mdx* mice. There is no statistical difference between both groups of animals. Values represent group means ± standard error (SE); n represents the number of animals in each group

**FIGURE 5:**
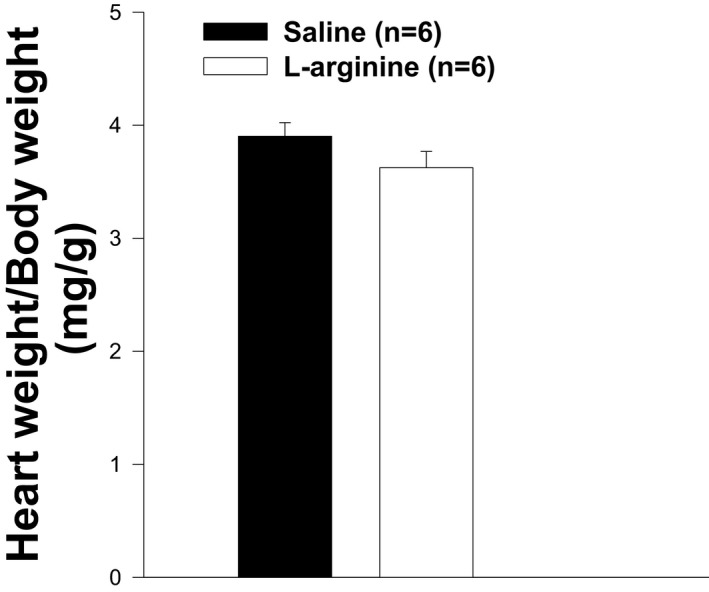
Heart weight normalized to body weight in L‐arginine and saline‐treated *mdx* mice. The ratios in both groups of animals are not different. There is no statistical difference between the two groups of animals. Values represent group means ± standard error (SE); n represents the number of animals in each group

**FIGURE 6:**
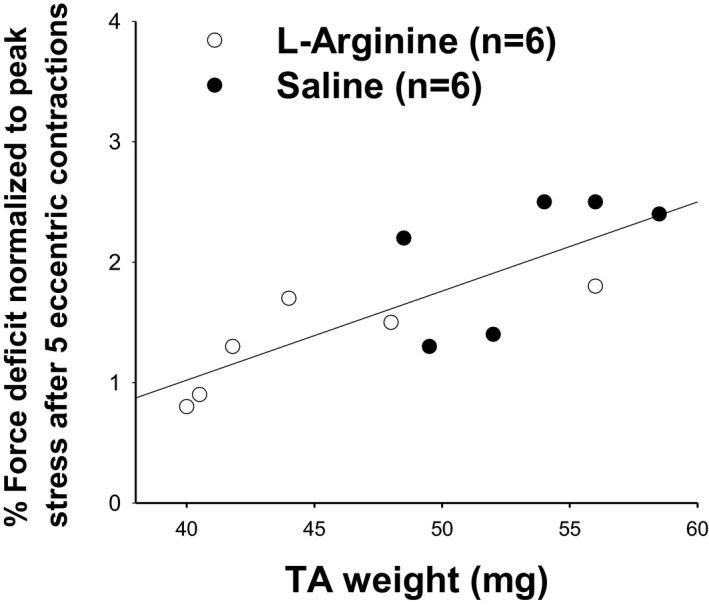
Relationship between the percentage of force deficit normalized to peak stress after five eccentric contractions and the muscle weight. Note a strong correlation between TA muscle weight and resistance to eccentric contraction after 6 weeks of treatment with 800 mg/kg of L‐arginine or saline (*r* = 0.758, *p = 0.011)

Skeletal muscles represent about 50% of total body mass, and DMD leads to progressive muscle destruction. We assumed that L‐arginine could impact body weight. As shown in Figure 1, body weights were not different between mice in both experimental groups. However, mice administered with L‐arginine showed a significant reduction in the weight of their TA muscle and heart when compared to those of control mice (Figures 2 and 3). When the TA muscle, and heart weights were normalized to the body weight there was no difference between mice in both experimental groups (Figures 4 and 5). Although there was no difference between mice when TA muscle weight was normalized to the body weight, we found a strong correlation (*r* = 0.758, *p* = 0.011) between TA muscle weight and resistance to eccentric contraction after 6 weeks of treatment with L‐arginine (Figure 6)

### 3.2 L‐Arginine decreased the level of central nucleation

**FIGURE 7:**
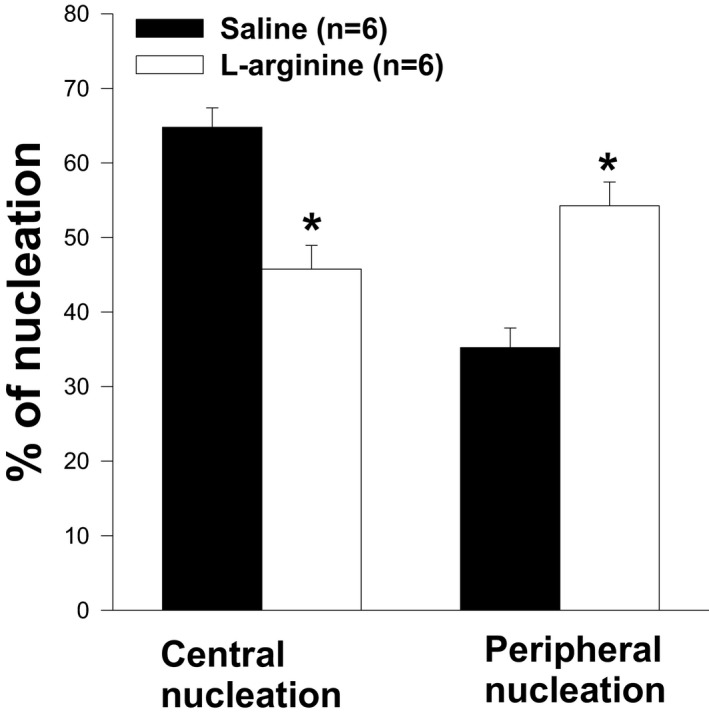
Percentage of nucleation in L‐arginine and Saline groups. The treatment with L‐arginine protects *mdx* muscle from muscle degeneration. Central nucleation in *mdx* mice treated with L‐arginine is significantly lower than in the control group (*p<0.05) while the peripheral nucleation in *mdx* mice treated with L‐arginine is significantly higher than in the control group (*p<0.05). Values represent group means ± SE; n represents the number of TA muscles analyzed

**FIGURE 8:**
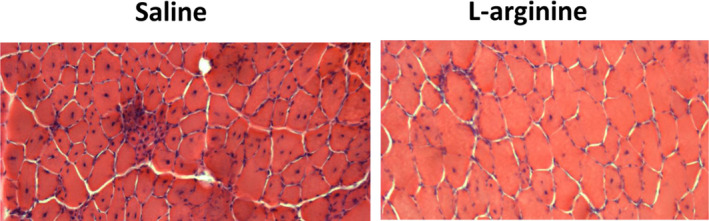
Representative images of transverse TA muscle sections stained with hematoxylin and eosin after 6 weeks of treatment with L‐arginine (right panel) or physiological saline solution (left panel) in *mdx* mice

**FIGURE 9:**
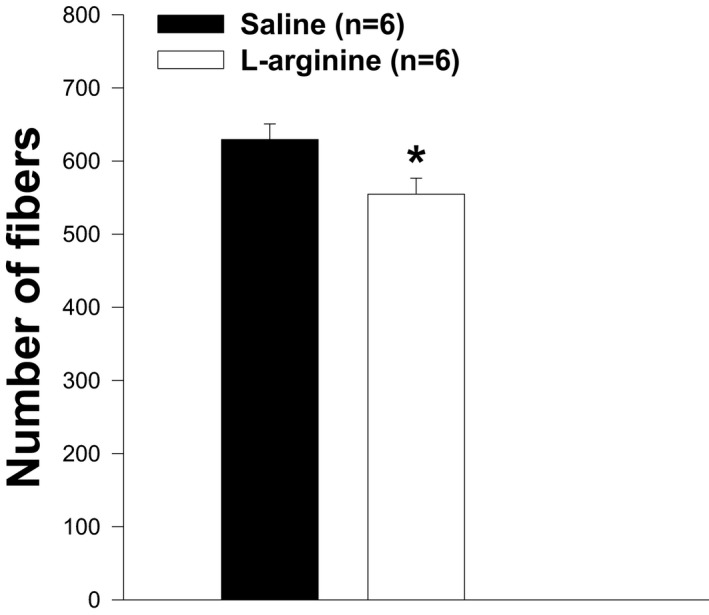
The number of muscle fibers in both groups of animals. The number of muscle fibers in *mdx* mice treated with L‐arginine is significantly lower than in the control group (*p<0.05), whereas the body weight is not different between the two groups of mice after 6 weeks of treatment. Values represent group means ± SE; n represents the number of TA muscles analyzed

**FIGURE 10:**
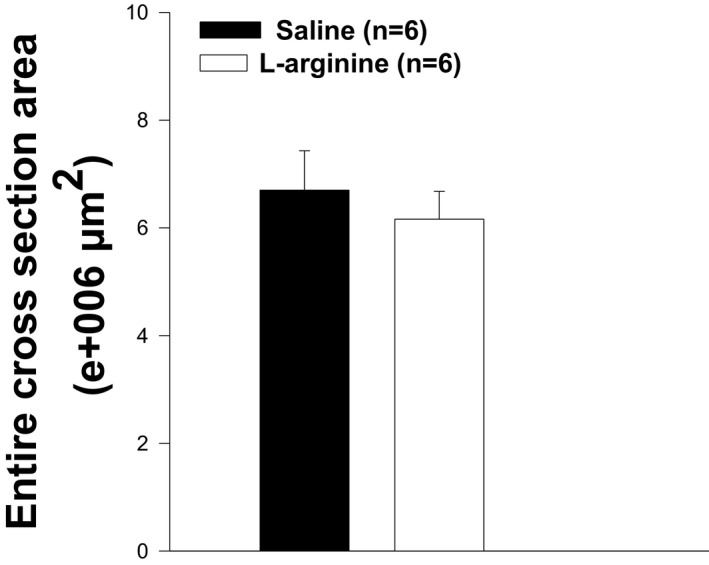
The entire cross‐sectional area measurement in L‐arginine and saline‐treated *mdx* mice. The entire cross area between the two groups of animals is not different. There is no statistical difference between the two groups of animals. Values represent group means ± standard error (SE); n represents the number of TA muscles in each group

The weight and level of central nucleation of TA muscles from wild‐type mice or dystrophin‐transfected *mdx* mice were found to be lower than those of *mdx* mice. To determine whether the low TA weight observed in L‐arginine‐treated *mdx* mice was associated with the low levels of central nucleation, H&E staining was performed on muscle sections. As shown in Figures 7 and 8, the level of central nucleation was significantly lower in TA muscles of L‐arginine‐treated mice compared to the control mice. It should be noted, however, that the percentage of peripheral nucleation was higher in L‐arginine‐treated mice than in saline‐injected mice (Figures 7). The number of fibers on the entire cross section was significantly higher in the saline group when compared with the L‐arginine group (Figures 9) even though the cross‐sectional areas were not statistically different (Figures 10).

### 3.3 L‐arginine did not affect the muscle contraction

**FIGURE 11:**
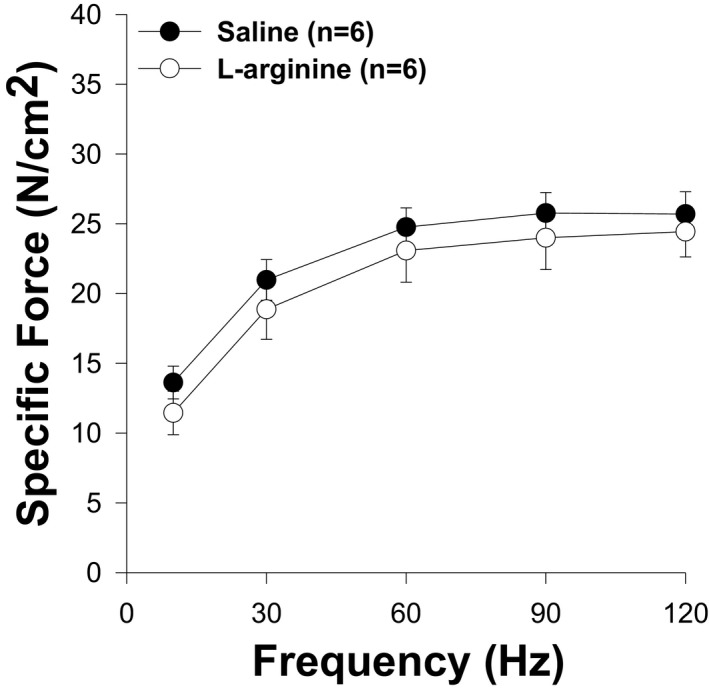
Effects of L‐arginine treatment on TA force‐generating capacity. Force–frequency relationship of TA in L‐arginine and control group is shown. There is no difference between the two groups of animals. Values represent group means ± SE; n represents the number of TA muscles analyzed

To determine whether central nucleation had an impact on the capacity of the muscle to generate force, we evaluated muscle contractility by electrical stimulation *in vivo*. As shown in Figure 11, the force‐generating capacity between both groups did not vary. However, the force‐generating capacity in wild‐type mice was significantly higher at all frequencies of stimulation compared to both groups of *mdx* mice (Figure S1).

### 3.4 L‐arginine increased the muscle resistance to mechanical‐stress induced injury

**FIGURE 12:**
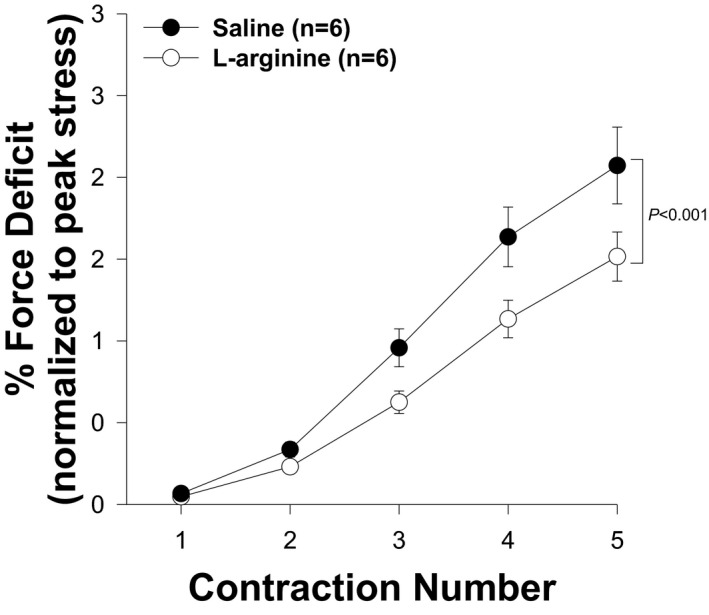
L‐arginine improves TA muscle resistance to eccentric contraction. Significant differences are detected after contraction number 2 (*p<0.05). Using ANOVA test, the force deficit observed during eccentric contractions is significantly lower in L‐arginine group relative to the control group (*p<0.01). Values represent group means ± SE; n represents the number of TA muscles analyzed

*Mdx* skeletal muscles were found to be abnormally susceptible to injury induced by mechanical stress.^3^, ^42^ To evaluate whether L‐arginine improves the ability of the TA muscle to resist mechanical stress, we performed five eccentric contractions and determined the magnitude of force deficits in the TA muscles between the two groups. As shown in Figure 12, L‐arginine significantly reduced the magnitude of the contraction‐induced force drop in *mdx* TAs muscle. Therefore, in addition to improved muscle histology, L‐arginine conferred significant functional benefits upon the muscles of *mdx* mice. It should be noted, however, that the TA muscle in wild‐type mice was more resistant than in the L‐arginine group (Figure S2).

### 3.5 Muscle resistance to eccentric contraction was not associated with utrophin overexpression

**FIGURE 13:**
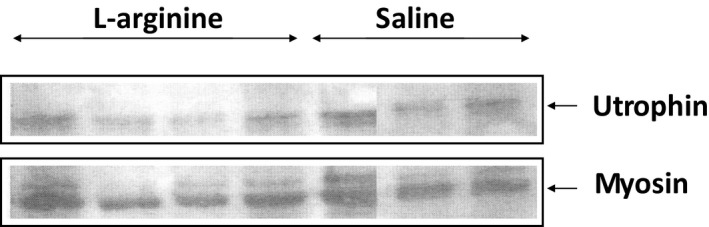
Representative western blot detection of utrophin in L‐arginine and saline‐treated *mdx* mice is shown

**FIGURE 14:**
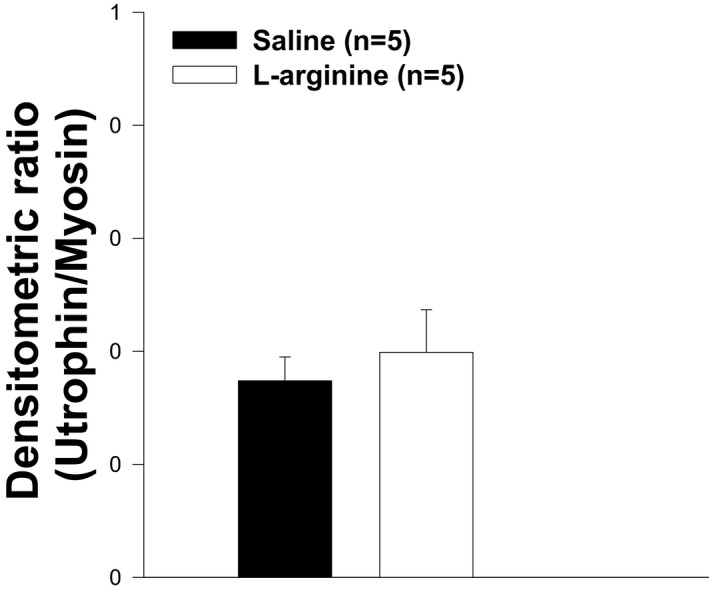
The scanning osf the band intensities from utrophin normalized to myosin is converted into histogram bars and allows comparison between TA muscles from L‐arginine and saline‐treated *mdx* mice. There is no statistical difference between both groups of animals. Data represent group means ± SE; n represents the number of TA muscles analyzed

**FIGURE 15:**
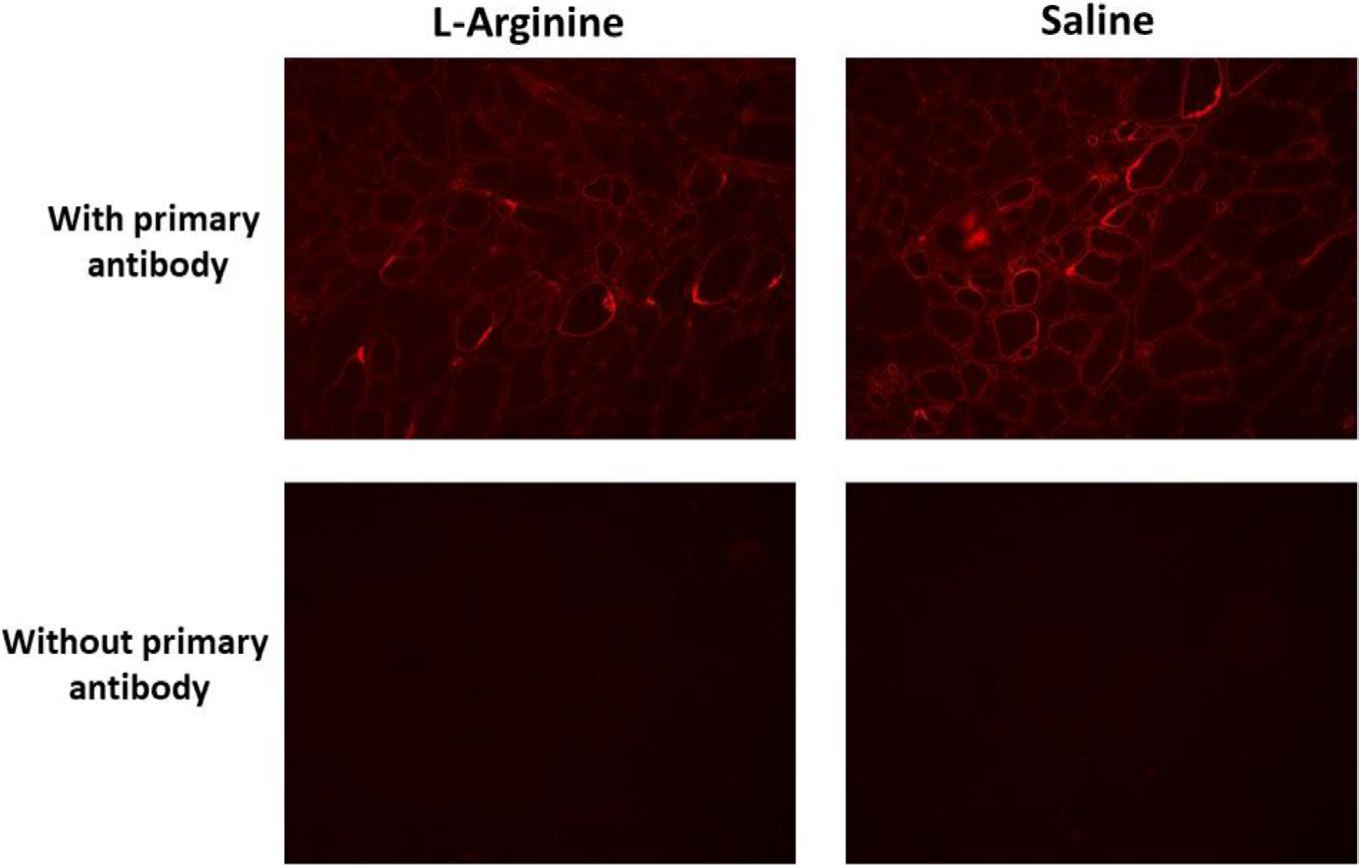
Immunofluorescence labeling of utrophin on control (right panel) and L‐arginine (left panel) treated TA muscles with a specific antibody directed against utrophin (upper panel). The lower panel represents the immunofluorescence labeling of utrophin on the same TA muscles without the antibody directed against utrophin protein

L‐arginine was found to induce utrophin upregulation in the skeletal muscles of *mdx* mice, resulting in an increased resistance in these muscles to mechanical stress induced by eccentric contractions. To determine whether the resistance of TA to eccentric contraction in L‐arginine‐treated *mdx* mice was associated with the upregulation of utrophin, immunostaining and immunoblotting were performed. As shown in Figure 13, western blot analyses, and the histogram representation of these results (Figure 14) provides clear evidence that the levels of utrophin expression did not vary between the two groups. These data suggest that the resistance of TA muscle to an eccentric contraction in the L‐arginine‐treated mice was not associated with the upregulation of utrophin. Furthermore, the immunostaining of utrophin did not show any evidence of utrophin upregulation in L‐arginine‐treated mice (Figure 15).

### 3.6 Effects of L‐arginine on creatine phosphate kinase activity

**FIGURE 16:**
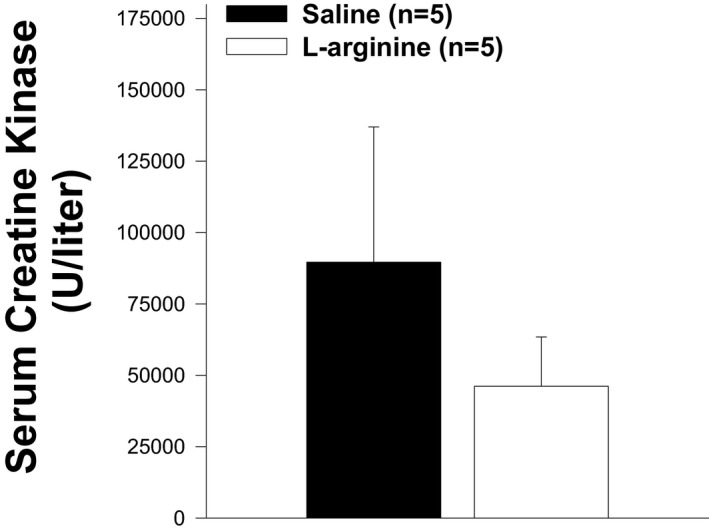
Effects of L‐arginine on serum creatine kinase. Serum creatine kinase release in L‐arginine and saline‐treated animals is shown. There is no statistical difference between both groups of animals. Values represent group means ± SE; n represents the number of animals in each group

Creatine phosphate kinase (CK) is an enzyme that is normally found inside myocytes. As such, CK in the blood is a sign of muscle injury. The levels of CK in the blood of *mdx* mice were found to be higher than in normal mice. To evaluate the levels of CK in *mdx* treated with L‐arginine or saline (control), serum CK was quantified after 6 weeks of the treatment. No statistical differences were observed between the two groups (Figure 16).

### 3.7 l‐arginine increased the nitric oxide (NO) production in TA muscle

**FIGURE 17:**
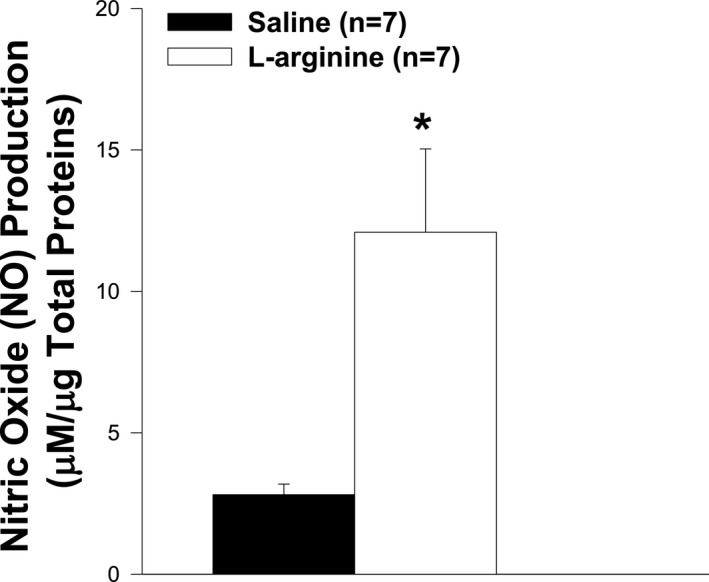
Impact of L‐arginine on nitric oxide (NO) production. NO production in *mdx* mice treated with L‐arginine is significantly higher than in saline‐treated mice (*p<0.05). Values represent group means ± SE; n represents the number of animals in each group

As shown above, the impact of L‐arginine on muscle weight, the level of central nucleation, and the resistance to eccentric contraction observed in TA were not associated with utrophin overexpression, as previously reported. To determine whether these improvements could be related to the production of NO, the levels of NO were measured. As shown in Figure 17, the production of NO in the L‐arginine group was significantly higher than in the control group.

## 4 DISCUSSION

In this manuscript, we investigated the early administration of L‐arginine in *mdx* neonatal mice as a therapeutic approach to delay the onset of DMD in TA muscle. Although the primary defect that gives rise to DMD has been known for a long time, an efficient therapy for the treatment of this disease has yet to be developed. To this end, several therapeutic approaches have been attempted, including molecular therapy, cellular therapy,^44^ and genetic therapy using plasmids^45^ and adeno and adeno‐associated (AAV) viruses to increase the expression of full‐length or shorter versions of dystrophin and utrophin.^46^, ^47^ Despite the success of these therapies in laboratories, further work is needed to translate these research findings into practical therapeutic strategies that can cure individuals suffering from DMD. A pharmacological treatment is another approach to treat individuals lacking dystrophin.^48^ Given its safety relative to other proposed approaches, pharmacological therapies that can provide even modest relief for individuals suffering from DMD remain a most attractive option. L‐arginine is one of the molecules which has been tested for this purpose and has been reported to improve the *mdx* mice phenotype via the upregulation of utrophin.^9^, ^10^ In this report, we demonstrated that L‐arginine ameliorates muscular dystrophy in *mdx* mice. However, this improvement was not associated with the upregulation of utrophin, as previously reported.

After the *in vivo* daily injection of L‐arginine in *mdx* neonatal mice, we observed an increase in the ability of TA muscle to resist to injuries caused by eccentric contractions, a major hallmark of dystrophin deficiency.^3^ We found that this beneficial effect is associated with an increase in NO production, rather than an upregulation of utrophin protein levels. Indeed, in our study, NO production was significantly higher in the L‐arginine group compared to the control group, while the level of utrophin expression did not vary between the different groups of mice. However, it is possible that different experimental conditions could be responsible for the discrepancy between our results and those reported in skeletal muscles from adult *mdx* mice treated with L‐arginine. Indeed, previous studies were performed on adult *mdx* mice (aged from 5 to 16 weeks), whereas our experiments involved neonatal *mdx* mice. It should be noted that the amount of utrophin obtained after L‐arginine treatment was variable and depended on either the age of the *mdx* mice, the duration of the L‐arginine administration, or the muscles studied.^9^, ^10^, ^49^ Another factor that could explain the discrepancy between our results and those of other groups on the skeletal muscles of *mdx* mice treated with L‐arginine is the concentration of L‐arginine. Indeed, the concentration of L‐arginine used in previous studies associated with utrophin upregulation varied from 200 to 400 mg of L‐arginine/kg of body weight,^9^, ^10^ whereas the concentration of L‐arginine used in our study was 800 mg of L‐arginine/kg of body weight.

The present results show that the increase in NO production in TA muscles was associated with a decrease in the levels of central nucleation in *mdx* mice treated with L‐arginine. A higher central nucleation level is a hallmark of skeletal muscle degeneration and regeneration in DMD.^3^ In our study, the levels of central nucleation in *mdx* mice treated with L‐arginine were significantly lower than in the control group. Our findings are consistent with previous studies that found that therapeutic approaches that increase NO and its key effector cGMP provide significant benefit to the dystrophic muscles of *mdx* mice.^50^, ^51^

In DMD, the absence of dystrophin leads to repeated cycles of skeletal muscle necrosis and regeneration with subsequent chronic inflammation.^52^ NO is generated by three types of nitric oxide synthase: nNOS, eNOS, and iNOS.^23^, ^29^, ^30^ iNOS is expressed by inflammatory cells and it is responsible for large production of NO during inflammatory responses.^53^, ^54^ In our study, we believe that the NO produced was less associated with inflammatory cells in the L‐arginine‐treated mice. In fact, it is TA muscles presenting the most regeneration (more central nucleation) that should produce more NO using iNOS pathway. In our study, however, TA muscles with less central nucleation produced more NO. Our findings are consistent with those that previously reported that the use of nitric oxide donors alone or combined with anti‐inflammatory drugs led to a reduction in muscle damage in *mdx* mice.^50^, ^55^ However, it must be emphasized that the increase in NO production in skeletal muscle can improve muscle regeneration, reduce muscle degeneration, or have no effect on central nucleation. This demonstrates the complexity of the relationship between NO and skeletal muscle regeneration.

In our study, we found that the number of muscle fibers was significantly lower in the L‐arginine group compared to the control group despite the fact the entire cross‐sectional area was not different between the groups of mice. This might have helped to minimize the amount of utrophin protein found in the L‐arginine group. However, it is not clear from the data presented here whether the lower number of muscle fibers found in the L‐arginine group has a direct effect on the amount of utrophin protein. The amount of utrophin obtained after L‐arginine treatment was variable and depended on either the age of the *mdx* mice, the duration of the L‐arginine administration, or the muscles studied as previously indicated.^9^, ^10^, ^49^ The muscle composition could explain the discrepancy between our results and those from the previous studies. Indeed, previous experiments were mainly performed on EDL and diaphragm muscles. We believe, however, that the TA muscle composition cannot be considered as an explanation for our findings. Indeed, Voisin et al. ^9^ reported 150% increase in utrophin in the TA muscle in response to L‐arginine versus the control group. In our study, the increase in utrophin after L‐arginine treatment was not statistically different from the one observed in the control group. As indicated above, this could be related to the lower number of muscle fibers in the L‐arginine group compared to the saline group. Similarly, the level of creatine kinase, in our study, was lower in the L‐arginine group compared to the control group but was not statistically different, which was not the case in previous studies on the same topic where the difference was significant. Although the difference in the amount of utrophin obtained in our experiment was not statistically significant between both treatment groups, we cannot completely rule out the potential role of utrophin as a mediator of the improvements against muscular dystrophy. Hence, these results suggest the importance of considering early L‐arginine treatment in babies diagnosed with DMD.

## 5 CONCLUSION

The results presented in this study demonstrate that L‐arginine improves the ability of TA muscle to resist mechanical stress and decreases the levels of central nucleation in *mdx* neonate mice. This improvement is more associated with an increase in the production of NO than with utrophin upregulation. Further studies will be needed to elucidate the mechanism by which L‐arginine improves muscular dystrophy. Nonetheless, our study strongly supports the use of L‐arginine as an attractive pharmacological therapeutic option at the onset of disease for the treatment of muscular dystrophy which should also be considered in pediatric populations.

## CONFLICT OF INTEREST

The authors declare that the research was conducted in the absence of any commercial or financial relationships that could be construed as a potential conflict of interest.

## AUTHORS’ CONTRIBUTIONS

GD wrote the manuscript. GD and RWRD performed the experiments. GD, RWRD, DSP, and ASC were involved in data processing, statistical analysis, and figures. The study was designed by GD, RWRD, DSP, and ASC. GD, RWRD, DSP, and ASC are involved in editing the manuscript. The submitted version of the manuscript was approved by all the authors.

## Supporting information

Fig S1‐S2

Fig S3‐S4

Fig S5

Fig S6

Fig S7‐S8

Fig S9

Fig S10
