# Supplementary material for: Early administration of L‐arginine in mdx neonatal mice delays the onset of muscular dystrophy in tibialis anterior (TA) muscle
Source: FASEB Bioadv. 2021 May 18;3(8):639–51. doi: 10.1096/fba.2020-00104 (PMC8332474; doi:10.1096/fba.2020-00104)
Supplement: Supplementary file 1 — Fig S1‐S2 [file FBA2-3-639-s006.pdf]

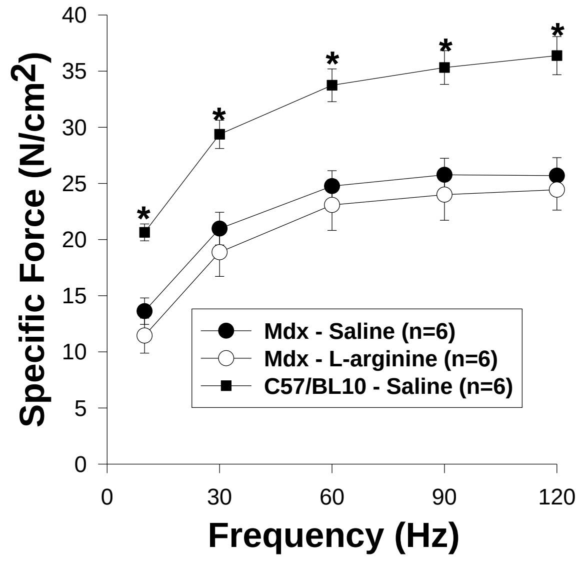

Suppl. Figure 1: Force-frequency relationship of TA muscle in two groups of mdx (L-arginine, and saline) and wild type is shown. There is no difference between the two groups of mdx mice. However, the force generating capacity is significantly higher in wild type mice (C57/BL10) compared to the mdx mice (*p<0.05). Values represent group means ± SE; n represents the number of TA muscles analyzed.

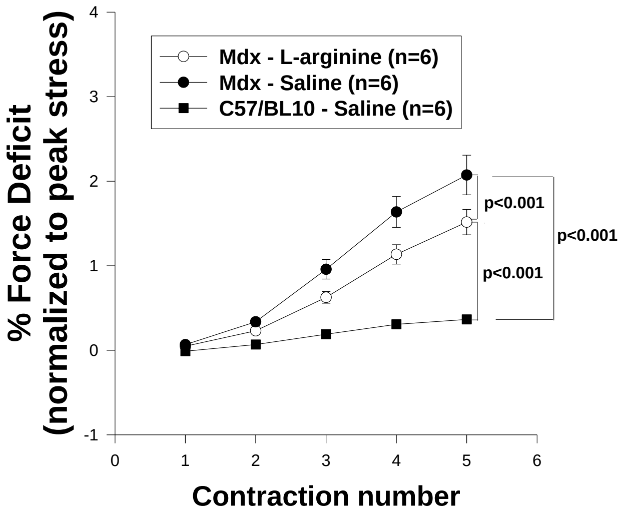

Suppl. Figure 2: TA muscle resistance to eccentric contraction. L-arginine improves *mdx* TA muscle resistance to eccentric contraction as shown. However, the TA muscle in wild type mice (C57/BL10) is significantly more resistant to eccentric contraction when compared to both groups of *mdx* mice. Significant differences are detected after contraction number 2 (*p<0.05). Using ANOVA test, the force deficit observed during eccentric contractions is significantly lower in wild type group relative to the two groups of *mdx* mice (*p<0.01) while L-arginine group it is significantly lower relative to the *mdx* treated with saline (*p<0.01). Values represent group means ± SE; n represents the number of TA muscles analyzed.
