# Supplementary material for: Early administration of L‐arginine in mdx neonatal mice delays the onset of muscular dystrophy in tibialis anterior (TA) muscle
Source: FASEB Bioadv. 2021 May 18;3(8):639–51. doi: 10.1096/fba.2020-00104 (PMC8332474; doi:10.1096/fba.2020-00104)
Supplement: Supplementary file 3 — Fig S5 [file FBA2-3-639-s002.pdf]

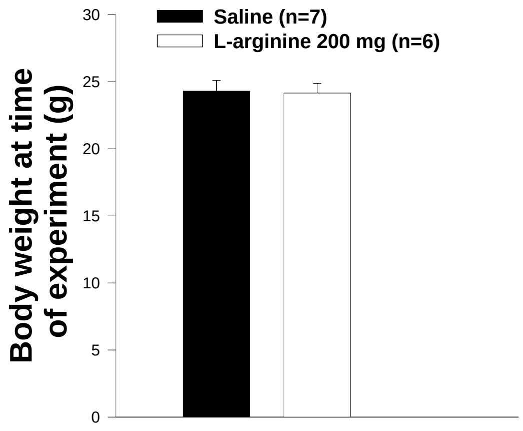

Suppl. Figure 5: Body weight in L-arginine (200 mg/kg) and saline treated *mdx* mice; There is no statistical difference between the two groups of animals. Values represent group means ± standard error (SE); n represents the number of animals analyzed.
