# Supplementary material for: Early administration of L‐arginine in mdx neonatal mice delays the onset of muscular dystrophy in tibialis anterior (TA) muscle
Source: FASEB Bioadv. 2021 May 18;3(8):639–51. doi: 10.1096/fba.2020-00104 (PMC8332474; doi:10.1096/fba.2020-00104)
Supplement: Supplementary file 5 — Fig S7‐S8 [file FBA2-3-639-s003.pdf]

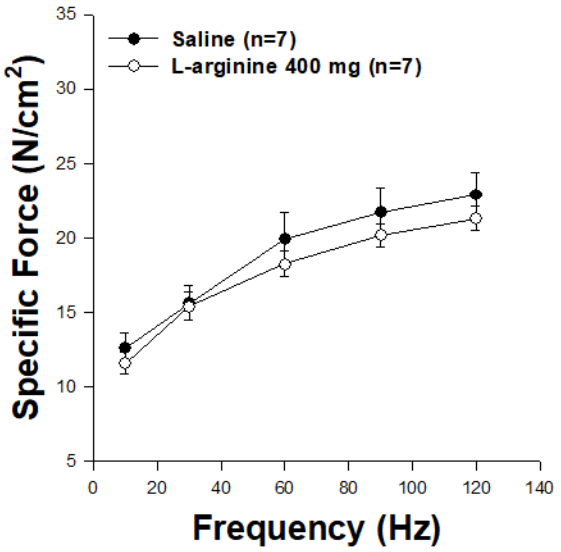

Suppl. Figure 7: Force-frequency relationship of TA muscle in the *mdx* group treated with L-arginine (400 mg/kg) and the saline group is shown. There is no difference between the two groups of *mdx* mice. Values represent group means ± SE; n represents the number of TA muscles analyzed.

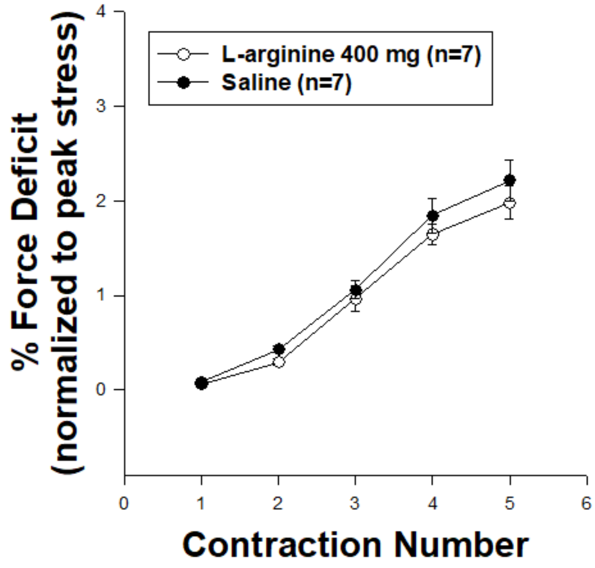

Suppl. Figure 8: TA muscle resistance to eccentric contraction in the *mdx* group treated with L-arginine (400 mg/kg) and the saline group is shown. There is no difference between the groups of mice. Values represent group means ± SE; n represents the number of TA muscles analyzed.
