# Supplementary material for: Early administration of L‐arginine in mdx neonatal mice delays the onset of muscular dystrophy in tibialis anterior (TA) muscle
Source: FASEB Bioadv. 2021 May 18;3(8):639–51. doi: 10.1096/fba.2020-00104 (PMC8332474; doi:10.1096/fba.2020-00104)
Supplement: Supplementary file 7 — Fig S10 [file FBA2-3-639-s005.pdf]

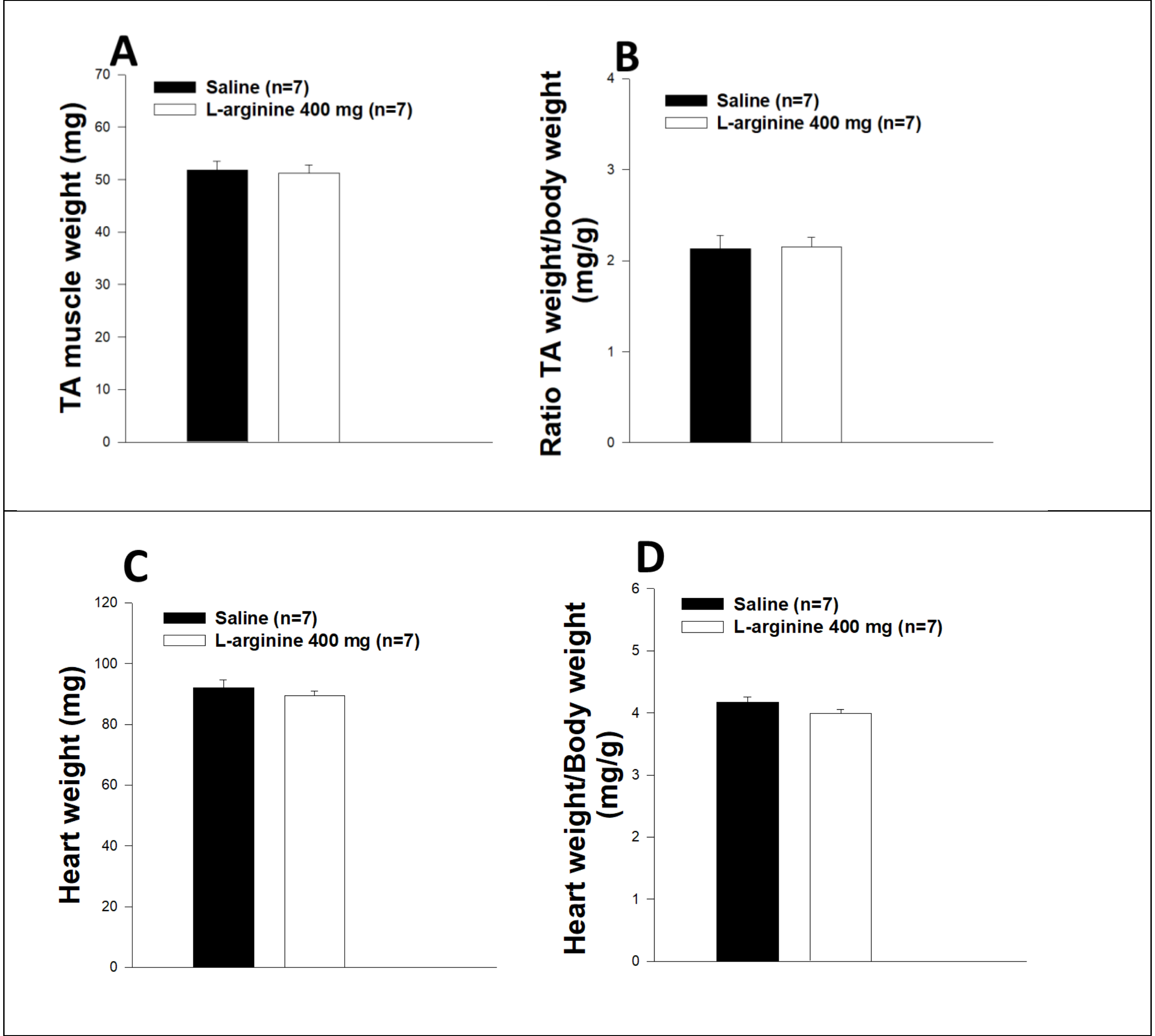

Suppl. Figure 10: **A)** TA muscle weight in L-arginine (400 mg/kg) and saline treated *mdx* mice; **B)** TA muscle weight normalized to the body weight in L-arginine and saline treated *mdx* mice. There is no statistical difference between the two groups of animals. Values represent group means ± standard error (SE); n represents the number of TA muscles analyzed; C**)** Heart weight in L-arginine (400 mg/kg) and saline treated *mdx* mice; **D)** Heart weight normalized to the body weight in L-arginine and saline treated *mdx* mice. There is no statistical difference between the two groups of animals. Values represent group means ± standard error (SE); n represents the number of hearts analyzed.
